# Clinical and Molecular Characterization of *PMP22* point mutations in Taiwanese patients with Inherited Neuropathy

**DOI:** 10.1038/s41598-017-14771-5

**Published:** 2017-11-10

**Authors:** Yi-Chu Liao, Pei-Chien Tsai, Thy-Sheng Lin, Cheng-Tsung Hsiao, Nai-Chen Chao, Kon-Ping Lin, Yi-Chung Lee

**Affiliations:** 1 Department of Neurology, Taipei Veterans General Hospital, Taipei, Taiwan, ROC; 2 Department of Neurology, National Yang-Ming University School of Medicine, Taipei, Taiwan, ROC; 3 Brain Research Center, National Yang-Ming University, Taipei, Taiwan, ROC; 4 Department of Neurology, College of Medicine, National Cheng Kung University, Tainan, Taiwan, ROC; 5 Department of Neurology, National Cheng Kung University Hospital, Tainan, Taiwan, ROC; 6 Division of Neurology, Department of Internal Medicine, Taipei Veterans General Hospital Taoyuan Branch, Taoyuan, Taiwan, ROC; 7 Graduate Institute of Physiology, College of Medicine, National Taiwan University, Taipei, Taiwan, ROC

## Abstract

Point mutations in the peripheral myelin protein 22 (*PMP22*) gene have been identified to cause demyelinating Charcot-Marie-Tooth disease (CMT) and hereditary neuropathy with liability to pressure palsy (HNPP). To investigate the mutation spectrum of *PMP22* in Han-Chinese population residing in Taiwan, 53 patients with molecularly unassigned demyelinating CMT and 52 patients with HNPP-like neuropathy of unknown genetic causes were screened for *PMP22* mutations by Sanger sequencing. Three point mutations were identified in four patients with demyelinating CMT, including c.256 C > T (p.Q86X) in two, and c.310delA (p.I104FfsX7) and c.319 + 1G > A in one each. One *PMP22* missense mutation, c.124 T > C (p.C42R), was identified in a patient with HNPP-like neuropathy. The clinical presentations of these mutations vary from mild HNPP-like syndrome to severe infantile-onset demyelinating CMT. *In vitro* analyses revealed that both *PMP22* p.Q86X and p.I104FfsX7 mutations result in truncated PMP22 proteins that are almost totally retained within cytosol, whereas the p.C42R mutation partially impairs cell membrane localization of PMP22 protein. In conclusion, *PMP22* point mutations account for 7.5% and 1.9% of demyelinating CMT and HNPP patients with unknown genetic causes, respectively. This study delineates the clinical and molecular features of *PMP22* point mutations in Taiwan, and emphasizes their roles in demyelinating CMT or HNPP-like neuropathy.

## Introduction

Mutations in the peripheral myelin protein 22 (*PMP22*) gene are the most common cause of inherited neuropathies, and different types of *PMP22* mutations lead to diverse phenotypes. Duplication of a 1.5-Mb DNA segment on chromosome 17p11.2-12 encompassing the *PMP22* gene causes Charcot-Marie-Tooth disease type 1 A (CMT1A)^1,2^, which is an autosomal dominant demyelinating neuropathy and the most common subtype of CMT. The same 1.5-Mb DNA segment triplication causes a more severe demyelinating polyneuropathy^3^, whereas large deletion at the same segment results in hereditary neuropathy with liability to pressure palsies (HNPP) characterized by episodic, recurrent sensory and motor mono-neuropathies at nerve entrapment sites^4,5^. In addition, a variety of point mutations in the *PMP22* gene has been identified in patients with a broad continuum of inherited neuropathies ranging from HNPP, CMT1E, infantile-onset severe dysmyelinating neuropathies similar to Dejerine-Sottas syndrome, to congenital hypomyelinating neuropathy^6–10^.

The protein encoded by the *PMP22* gene is a glycoprotein of 160 amino acids and constitutes 2–5% of overall peripheral myelin proteins^11,12^. PMP22 protein forms a predicted structure of four transmembrane domains, two extracellular loops, and cytoplasmic N- and C-terminal tails^11,12^. The homophilic adhesion between two PMP22 proteins and the heterophilic interaction between PMP22 and myelin protein zero (P0) protein on the opposing membranes of two Schwann cells are essential for the compactness and stability of peripheral myelin^13,14^. Besides, PMP22 protein also modulates the proliferation and apoptosis of Schwann cells^15–18^. More than 60 different mutations in the *PMP22* gene have been reported to date^6^; however, a majority of them have not been functionally validated by *in vitro* analyses and studies about *PMP22* point mutations in Chinese populations remain sparse. The aim of present study is to investigate the frequency and spectrum of *PMP22* point mutations in cohorts of Taiwanese patients with CMT or HNPP-like neuropathy. The clinical and molecular features of the identified *PMP22* mutations were also characterized.

## Results

### Identification of the *PMP22* point mutations

**Figure 1:**
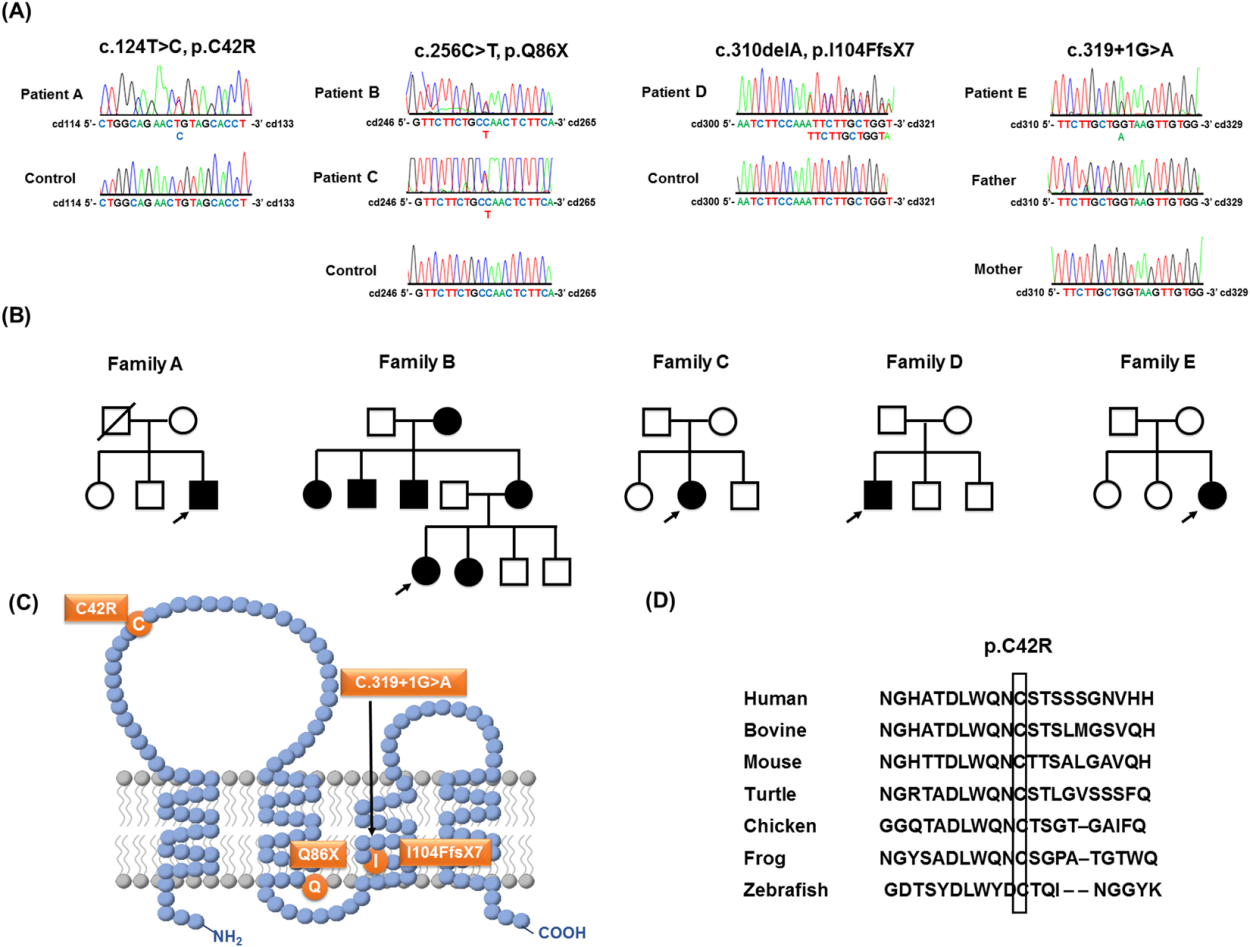
The *PMP22* mutations and the pedigrees harboring the *PMP22* mutations in this study. (**A**) Sanger sequencing traces demonstrating the *PMP22* c.124 T > C (p.C42R), c.256 C > T (p.Q86X), c.310delA (p.I104FfsX7) and c.319 + 1G > A mutations. (**B**) The five pedigrees harboring the *PMP22* mutations. Open symbol: unaffected; filled symbol: affected; symbol with a diagonal line: deceased; arrow: proband. (**C**) Schematic representation of the PMP22 protein structure and the positions of the four *PMP22* mutations. (**D**) The p.C42R mutation resides in an evolutionarily conserved region, as shown by the alignment of multiple PMP22 orthologs from various species.

Fifty-three patients of unknown genetic diagnosis were selected from 265 unrelated individuals with demyelinating CMT in whom 175 have *PMP22* duplication, 26 have a mutation in the gap junction protein beta 1 (*GJB1*) gene and 11 have a mutation in the myelin protein zero (*MPZ*) gene. Another group of 52 molecularly unassigned patients were recruited from 138 index patients with a HNPP-like phenotype, after *PMP22* deletion was excluded. Mutational analyses of *PMP22* in the 53 patients with demyelinating CMT revealed three point mutations, including c.256 C > T (p.Q86X) in two patients, and c.310delA (p.I104FfsX7) and c.319 + 1G > A in one patient each (Fig. 1A and B). One of the 52 patients with HNPP-like phenotype was found to carry a *PMP22* missense mutation, c.124 T > C (p.C42R). All the five patients harboring a *PMP22* point mutation are heterozygous for the mutation.

Among these four mutations, the p.C42R mutation alters the amino acid residue residing in the extracellular loop of PMP22 protein and the other three mutations affect the amino acid sequence of PMP22 transmembrane domains (Fig. 1C). The c.256 C > T (p.Q86X) and c.319 + 1G > A mutations have been recognized to be pathogenic for CMT1 before^19,20^, whereas the c.310delA (p.I104FfsX7) mutation is novel. The c.124 T > C (p.C42R) mutation was lately reported by a study analyzing patients with inherited neuropathies by a targeted gene panel without clear phenotype information and functional validation^21^. The pathogenicity of the c.310delA (p.I104FfsX7) is evident by the fact that *PMP22* c.312delT (p.I104IfsX7) mutation, of which putative protein product is almost identical to that produced by the c.310delA mutation, has been shown to be a pathogenic mutation for CMT1^20^. The c.124 T > C (p.C42R) was found in an apparently sporadic patient (Fig. 1B), whose families’ sample was unavailable for co-segregation analysis. This mutation is not present in the 141,352 individuals with diverse ethnicities in the genome Aggregation Database (gnomAD) that includes approximately 9300 individuals from East Asian populations. It is also absent in the dbSNP database and 1497 ethnically matched control individuals. The 42th amino acid residue of human PMP22 protein is highly evolutionarily conserved (Fig. 1D). Additionally, the pathogenicity of the c.124 T > C (p.C42R) mutation is supported by *in silico* analyses using three different programs—Polyphen2, Mutation Taster and CADD. Polyphen2 predicts *PMP22* c.124 T > C (p.C42R) to be probably damaging with a score 0.999 and false positive rate 0.01^22^. Mutation Taster predicts this variant to be disease causing with a very strong probability value, 0.9999, which indicates a high “security” of the prediction^23^. The CADD v1.2 PHRED score for *PMP22* c.124 T > C (p.C42R) is 25.7, which places this variant in the top 0.27% most deleterious variants in the genome^24^.

### Clinical features of patients harboring the *PMP22* point mutations

The clinical characteristics of the five patients with *PMP22* point mutations are summarized in Table 1. Patient A, who is heterozygous for the *PMP22* p.C42R mutation, developed episodic numbness in his palms while holding things since age 20 years. Labored work evoked recurrent, transient numbness and soreness in his legs since age 35 years. Neurological examinations at age 40 years demonstrated diffusely diminished deep tendon reflexes, but there was neither weakness nor atrophy over limb muscles. All modalities of sensation were preserved. The nerve conduction studies (NCS) showed generalized prolonged distal latencies and F-wave latencies of the motor or sensory nerves and mildly decreased motor nerve conduction velocities (Table 2).

**Table 1:** Clinical manifestations of the patients with *PMP22* point mutations.

Patient	A	B	C	D	E
*PMP22* mutation	c.124 T > C, p.C42R	c.256 C > T, p.Q86X	c.256 C > T, p.Q86X	c.310delA, p.I104FfsX7	c.319 + 1G > A
Sex	Male	Female	Female	Male	Female
Age at onset (y)	20	Teenage	20	< 1 y	19
Age at exam (y)	40	28	29	26	20
Clinical diagnosis	HNPP	Demyelinating CMT	Demyelinating CMT	Demyelinating CMT	Demyelinating CMT
Inheritance	Apparently sporadic	Autosomal dominant	Apparently sporadic	Apparently sporadic	*de novo*
First symptom	Left hand numbness	Foot drop	Foot drop	Delayed walking	Left hand numbness
Muscle strength (MRC scale)					
Dorsiflexion	5	0	0	0	5
Plantar flexion	5	2	2	1	5
Knee flexion	5	4	4	4	5
Thumb abduction	5	3	4	3	5
Wrist extension	5	4	5	4	5
Muscle atrophy	Nil	Distal UL + LL	Distal UL + LL	Distal UL + LL	Distal LL
Knee DTR (Rt/Lt)	+/+	−/−	−/−	−/−	−/−
Ankle DTR (Rt/Lt)	−/+	−/−	−/−	−/−	−/−
Sensory loss	Nil	Distal to ankles	Toes and distal fingers	Distal to ankles and wrists	Nil
References	Laššuthová *et al*.^21^	Numakura *et al*.^19^	Numakura *et al*.^19^	This study	Nelis *et al*.^20^

Abbreviation: HNPP = hereditary neuropathy with liability to pressure palsies; CMT = Charcot-Marie-Tooth disease; MRC = Medical Research Council; LL = lower limbs; UL = upper limbs; DTR = deep tendon reflex; Rt = right; Lt = left.

**Table 2:** Nerve conduction studies of patients with *PMP22* point mutations.

Patient	A	B	C	D	E
*PMP22* mutation	c.124 T > C, p.C42R	c.256 C > T, p.Q86X	c.256 C > T, p.Q86X	c.310delA, p.I104FfsX7	c.319 + 1G > A
Median nerve					
DML (ms)	8.3	NR	NR	18.2	10.8
MNCV (m/s)	42.0	NR	NR	4	21.6
CMAP (mV)	10.2	NR	NR	0.4	4.4
F-wave L (ms)	36.3	NR	NR	NR	NR
DSL (ms)	3.9	NR	NR	NR	NR
SNAP (uV)	10	NR	NR	NR	NR
Ulnar nerve					
DML (ms)	5.8	18.2	22.1	NR	7.0
MNCV (m/s)	48.9	5.9	7.6	NR	20.5
CMAP (mV)	7.5	0.4	0.2	NR	6.1
F-wave L (ms)	37.6	NR	NR	NR	NR
DSL (ms)	3.9	NR	NR	NR	NR
SNAP (uV)	10	NR	NR	NR	NR
Tibial nerve					
DML (ms)	5.4	NR	NR	NR	NR
MNCV (m/s)	34.9	NR	NR	NR	NR
CMAP (mV)	11.5	NR	NR	NR	NR
F-wave L (ms)	61.8	NR	NR	NR	NR
Sural nerve					
DSL (ms)	4.5	NR	NR	NR	NR
SNAP (uV)	12.6	NR	NR	NR	NR

Abbreviation:

DML = distal motor latency; MNCV = motor nerve conduction velocity; CMAP = compound motor action potential amplitude; F-wave L = F-wave latency; DSL = distal sensory latency; SNAP = sensory nerve action potential (antidromic); NR = no response.

Normal values:

Median nerve: DML ≤ 4.4 ms; MNCV ≥ 51.9 m/s; CMAP ≥ 6.4 mV; F-wave L ≤ 29 ms; DSL ≤ 3.2 ms; SNAP ≥ 17μV.

Ulnar nerve: DML ≤ 3.5 ms; MNCV ≥ 56.1 m/s; CMAP ≥ 7.0 mV; F-wave L ≤ 29 ms; DSL ≤ 3.0 ms; SNAP ≥ 8 μV.

Tibial nerve: DML ≤ 6.4 ms; MNCV ≥ 42.9 m/s; CMAP ≥ 4.1 mV; F-wave L ≤ 53 ms;

Sural nerve: DSL ≤ 3.5 ms; SNAP ≥ 12 μV.

Patient B, who has the *PMP22* p.Q86X mutation, suffered from a slowly progressive weakness and atrophy in the distal limbs since teenage. She had a normal developmental milestone on walking. Her sister, mother and another four of her maternal relatives also had a similar clinical phenotype. Physical examinations at age 28 revealed generalized weakness over four limbs with predominant involvement of the lower limbs and distal limb muscles, severe atrophy of the muscles in the legs and feet, generalized areflexia, and stock and glove pattern of sensory loss in the regions below ankles. Patient C, also carrying the *PMP22* p.Q86X mutation, presented with foot drop as the initial manifestation and then developed slowly progressive weakness and atrophy of the distal limb muscles since age 20 years. She had a normal onset of walking and denied any family history of neuromuscular diseases. Neurological examinations at age 29 demonstrated severe atrophy and weakness of the muscles in the legs and feet (score 2/5 and 0/5 on the Medic Research Council scale, respectively), mild weakness of the thigh muscles and intrinsic hand muscles (score 4/5), generalized areflexia, and sensory loss in the toes and distal fingers. For both patients carrying the *PMP22* p.Q86X mutation, the NCS revealed a severe demyelinating polyneuropathy with a single digit ulnar motor nerve conduction velocity (Table 2).

Patient D is heterozygous for the *PMP22* p.I104FfsX7 mutation. The patient had delayed motor milestones and was not able to walk by himself till 3 years of age. He could never run, jump or walk well. He had difficulties in buttoning and opening a jar at age 7 years and experienced slowly progressive weakness, atrophy and sensory loss in the distal limbs since age 18 years. Neurological examinations at age 26 years revealed hammer toes, severe atrophy and weakness of tibialis anterior, gastrocnemius and foot muscles (score 0–1/5), mild weakness of the thigh muscles (score 4/5), atrophy and weakness of the intrinsic hand muscles (score 3/5), generalized areflexia, and sensory loss in the regions distal to ankles and wrists with a positive Romberg’s test (see Supplementary video and Supplementary Fig. S1). The NCS showed a severe demyelinating polyneuropathy with a median motor nerve conduction velocity of 4 m/s (Table 2). He denied any relevant family history.

Patient E, who harbored a *de novo PMP22* c.319 + 1G > A mutation, experienced two episodes of transient numbness over left 4, 5^th^ digits lasting for 2 months each at age 19 years. She could still play tennis well at age 20 years. Physical examinations at age 20 revealed pes cavus, hammer toes, generalized areflexia, mild weakness of bilateral extensor digitorum brevis and no sensory loss. The NCS revealed a demyelinating polyneuropathy with a median motor nerve conduction velocity of 21.6 m/s (Table 2). Her parents were healthy and did not carry the *PMP22* mutation.

### *In vitro* analyses of PMP22 expression

**Figure 2:**
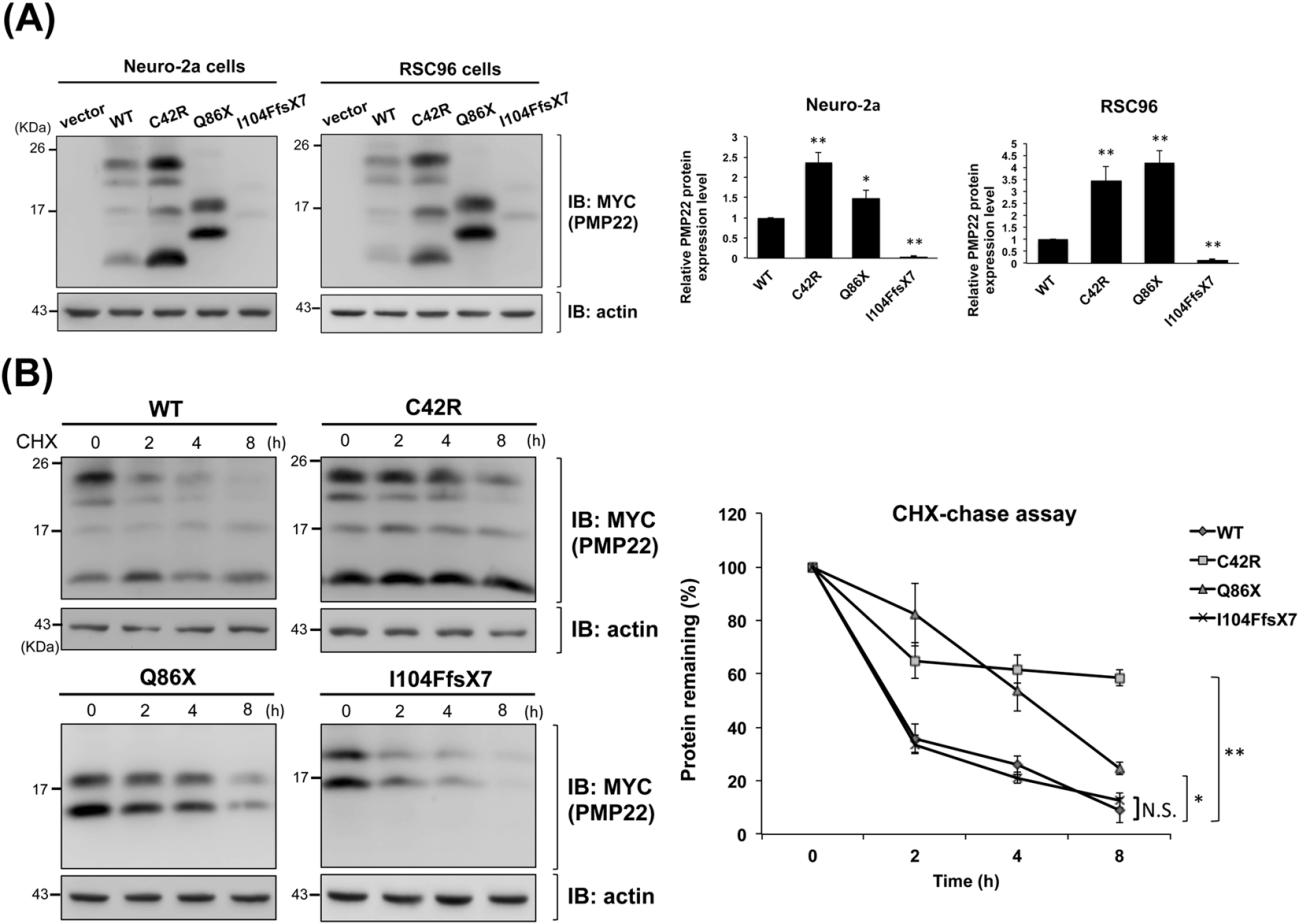
*In vitro* characterization of the wide-type (WT), C42R, Q86X, and I104FfsX7 mutant PMP22 proteins. (**A**) Representative Western blot analysis and densitometric quantification of steady-state PMP22 expression in the Neuro-2a cells and RSC96 cells. Cells were transfected with WT or mutant *PMP22* expression plasmids. Actin was used as a loading control. The error bars indicate standard error of the mean (SEM) from 3 independent experiments. The asterisks indicate statistically significant differences (* p < 0.05, ** p < 0.01). (**B**) Analyses of the stability of the WT and mutant PMP22 proteins. Neuro-2a cells were transfected with WT or mutant *PMP22* expression plasmids for 48 hours and then subjected to cycloheximide (CHX)-chase assays. Representive Western blots are shown. All values are shown as means ± SEM (n = 3). The asterisks indicate statistically significant differences (* p < 0.05, ** p < 0.01, NS = not significant).

To investigate the molecular consequences of the *PMP22* mutations identified in this study, we first cloned the wide-type (WT) *PMP22* cDNA into pcDNA3.1/myc-His vector and then introduced the p.C42R, p.I104FfsX7, and p.Q86X mutations into WT *PMP22* expression plasmids separately. Mouse neuroblastoma cell line (Neuro-2a) and two kinds of rat neuronal Schwannoma cell lines (RSC96 and RT4-D6P2T) were used in the *in vitro* functional studies. Cells were transfected with WT plasmids or either one of the mutant constructs to investigate functional consequences of these mutant PMP22 proteins. The expression levels of WT or mutant PMP22 protein at steady-state were measured by Western blotting. The p.Q86X and p.I104FfsX7 mutations resulted in a smaller, truncated protein product. The I104FfsX7 mutant protein was expressed at a significantly lower level than achieved with WT PMP22 protein (Fig. 2A, p < 0.001 for both Neuro-2a cells and RSC96 cells). Interestingly, we also observed substantial protein signal of the I104FfsX7 mutant at the interface between stacking gel and separating gel (see Supplementary Fig. S2), suggesting that the mutant protein got aggregated and thus tended to be stuck at the gel interface. Additionally, in both Neuro-2a and RSC96 cell lines, the steady-state protein levels of C42R and Q86X mutant PMP22 were significantly greater than that of WT PMP22 (Fig. 2A, all p < 0.05). We next evaluated the influence of these mutations on PMP22 degradation by a cycloheximide (CHX)-chase assay. The p.C42R and p.Q86X mutations attenuated PMP22 protein degradation (Fig. 2B and Supplementary Fig. S3), which might be responsible for the higher steady-state levels of these two mutant proteins.

**Figure 3:**
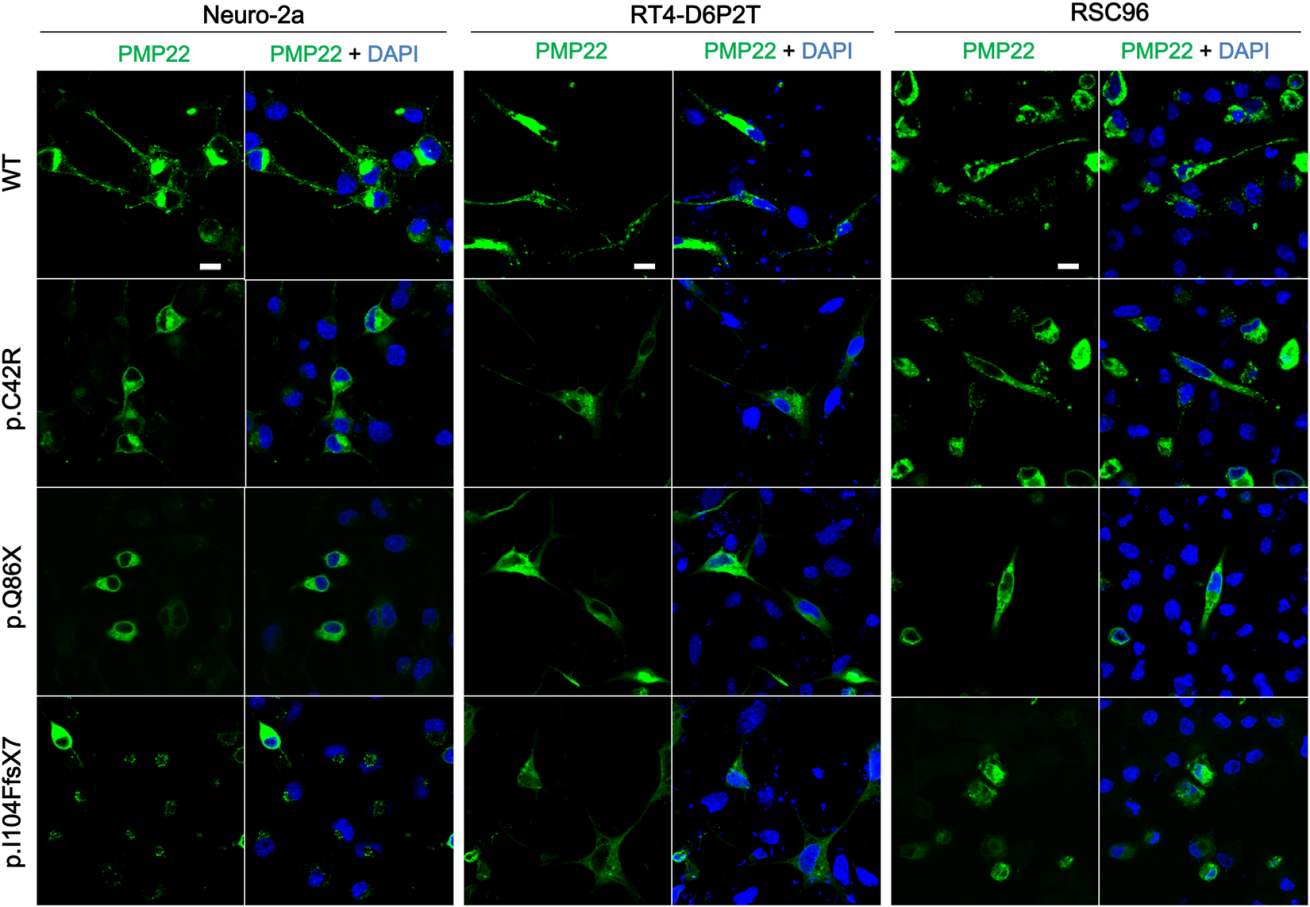
Immunofluorescence analyses of wide-type (WT) and mutant PMP22 proteins in the transfected Neuro-2a, RT4-D6P2T, and RSC96 cells. Confocal fluorescence images of transfected cells labeled with an Alexa Flour 488-conjugated anti-c-Myc antibody for detecting exogenous myc-tagged PMP22 proteins (green). Cells expressing WT or C42R PMP22 had a clear PMP22-specific staining on the cell membrane, but cells expressing C42R PMP22 had a much greater portion of PMP22-specific staining in the cytoplasm than cell membrane. Cells expressing Q86X or I104FfsX7 PMP22 showed an abundant PMP22-specific reticular staining throughout the cytosol but scarcely present on the cell membrane. Cell nuclei were counterstained with 4′,6-diamidino-2-phenylindole (DAPI, blue). Scale bar = 10 um.

PMP22 is a transmembrane protein and a proper cell membrane localization is critical for PMP22 protein to maintain myelin integrity; therefore, we conducted immunofluorescence analyses to visualize the myc-tagged WT or mutant PMP22 proteins in three cell lines with neuronal background. Cells expressing WT or C42R PMP22 had a clear PMP22-specific staining on the cell membrane, but cells expressing C42R PMP22 had a much greater portion of PMP22-specific staining in the cytoplasm than cell membrane (Fig. 3). For cells expressing Q86X or I104FfsX7 PMP22, the PMP22-specific reticular staining was abundant throughout the cytosol but scarcely present on the cell membrane (Fig. 3). And not surprisingly, numerous small aggregates were found in Neuro-2a cells expressing I104FfsX7 PMP22. Similar results were also found in both RSC96 and RT4-D6P2T cell lines expressing the I104FfsX7 mutant, although the aggregates tended to be smaller and fewer in these two cell lines. These results are consistent with the western blot detection of I104FfsX7 PMP22 aggregates.

## Discussion

To understand the contribution of *PMP22* point mutations to inherited neuropathy in Taiwan and their clinical and genetic features, we screened patients with demyelinating CMT or HNPP-like phenotype for mutations in the *PMP22* gene using Sanger sequencing. We identified four disparate *PMP22* point mutations in five unrelated patients with variable clinical presentations from mild HNPP-like syndrome to severe infantile-onset demyelinating CMT. Among these mutations, the p.C42R mutation was associated with a mild HNPP phenotype and partially affected intracellular trafficking of PMP22 protein to cell membrane in *in vitro* studies. The p.Q86X mutation, which exhibited a more severe phenotype, resulted in a truncated protein product and significantly impaired cell membrane localization of PMP22 protein. The p.Q86X mutation was found in two unrelated patients with similar clinical manifestations, presenting as a young adulthood-onset, progressive and disable polyneuropathy. In addition, the p.I104FfsX7 mutation linked to an infantile-onset severe polyneuropathy led to a truncated PMP22 with a very low expression level, insoluble cytoplasmic aggregate formations, and the failure of cell membrane localization. The c.319 + 1G > A mutation was identified in a 20-year-old lady with a very mild symptom but severe electrophysiological abnormalities. The wide clinical spectrum in the patients with *PMP22* point mutations indicates that *PMP22* sequencing should be considered in all of the molecularly unassigned patients with inherited demyelinating neuropathy or HNPP-like syndrome, regardless of their clinical severities.

Among the four *PMP22* mutations identified in this study, the pathogenicity of the p.Q86X, p.I104FfsX7 and c.319 + 1G > A mutations in CMT have been supported by previous reports^19,20^. However, the causal role of p.C42R mutation in inherited neuropathies has not been clearly demonstrated yet. The present study further supported the pathogenicity of *PMP22* p.C42R mutation by the following evidences. First, this mutation was identified in one of 52 unrelated patients with HNPP-like phenotype, but not in any of the 1,497 Taiwanese control individuals. The significant discrepancy in the prevalence of this mutation between patients and controls sustains the association between the *PMP22* p.C42R mutation and HNPP (Fisher’s exact test, p value = 0.034). Second, this mutation is absent in the gnomAD, the large database containing 126,216 exome sequences and 15,136 whole-genome sequences from different ethnic groups. Third, the p.C42R mutation occurs at an evolutionarily conserved amino acid residue of the human PMP22 protein and multiple computational predictive programs support its pathogenic effect. Forth, multiple *PMP22* missense mutations have also been reported to cause HNPP. Accordingly, the p.C42R mutation is classified as a likely pathogenic variant based on the guidelines for the interpretation of sequence variants recommended by the American College of Medical Genetics and Genomics and the Association for Molecular Pathology^25^. *PMP22* point mutation is a rare cause of inherited neuropathy. Previous studies showed that *PMP22* point mutations account for 0.6–1.4% of total CMT cases in Caucasian populations^26–29^. According to our findings, the prevalence of *PMP22* point mutations in Taiwanese CMT is also within this range. We identified *PMP22* point mutations in four out of 53 molecularly unassigned demyelinating CMT patients selected from 265 demyelinating CMT patients or 382 patients with all kinds of CMT, indicating that *PMP22* point mutations are responsible for 1.5% (4/265) of demyelinating CMT and 1.0% (4/382) of total CMT in Taiwan. Despite with a low prevalence, the *PMP22* point mutations remain to be an important cause of demyelinating CMT without common mutations because they explain for 7.5% (4/53) of such cases.

Only 68–84% patients with a HNPP-like neuropathy are attributed by a 1.5-Mb DNA segment deletion on *PMP22* ^30–32^. Frameshift, nonsense, or missense mutations on the *PMP22* gene are also well-known causes of HNPP^6^; however, clinical manifestations and electrophysiological features are not useful to distinguish patients with *PMP22* point mutation from those with *PMP22* deletion^31^. We found a *PMP22* point mutation in one out of 52 HNPP-like neuropathy patients with unclear causes, whom were chosen from 138 patients with HNPP-like phenotype after excluding *PMP22* deletion. Therefore, *PMP22* point mutations only accounted for 0.7% (1/138) of overall HNPP-like neuropathy and 1.9% (1/52) of HNPP-like neuropathy with unknown genetic diagnosis in Taiwan. Additional genetic contributions of HNPP-like neuropathy in Han-Chinese population await further investigation. The other possible causes of HNPP-like neuropathy include mutations on the septin 9 (*SEPT9*) gene that causes hereditary neuralgic amyotrophy^33^, a heterozygous *MPZ* p.Y145X mutation^34^, and a novel genetic locus on chromosome 21q21 implicated in a hereditary recurrent neuropathy^35^.

The molecular pathogenic mechanism of *PMP22* point mutations might differ from those of CMT1A with a *PMP22* duplication and HNPP with a *PMP22* deletion. The gene dosage effect of *PMP22* has been proposed to explain the phenotypic differences between CMT1A and HNPP, based on the facts that *PMP22* mRNA and protein levels in the peripheral nerve system are increased in CMT1A but decreased in HNPP. In the cellular transfection studies, we demonstrated that the *PMP22* p.Q86X and p.I104FfsX7 mutations, which are separately associated with young adulthood-onset and infantile-onset demyelinating CMT, result in almost total cytoplasmic retention and loss of cell membrane localization of PMP22. These findings are different from the increased PMP22 expression in CMT1A. We also revealed that the HNPP-causing *PMP22* p.C42R mutation had a mildly defective intracellular trafficking of PMP22 to cell membrane. Similar to previous studies, our findings affirm that cytoplasmic retention and impaired cell membrane localization of PMP22 are common mechanisms underlying the pathology of CMT1A/HNPP due to *PMP22* point mutations^36,37^.

In conclusion, *PMP22* point mutations are uncommon causes of demyelinating CMT and HNPP-like neuropathy. However, it still accounts for 7.5% of molecularly unassigned demyelinating CMT in the absence of *PMP22* duplication, *GJB1* mutations or *MPZ* mutations, as well as 1.9% of HNPP-like neuropathy of unknown genetic causes in Taiwan. This study delineates the clinical and molecular features of *PMP22* point mutations in Taiwan, expands the spectrum of *PMP22* point mutations, and emphasizes its role in demyelinating CMT and HNPP-like neuropathy.

## Methods

### Patients

Fifty-three individuals with demyelinating CMT of unknown genetic diagnosis were enrolled into this study. These patients were selected from a consecutive series of 382 unrelated patients with CMT at the Neurology Clinics of Taipei Veterans General Hospital, of whom 265 patients have demyelinating polyneuropathy ascertained by standard clinical and electrophysiological evaluations. Median or ulnar nerve motor nerve conduction velocity with a cutoff value of 38 m/s is used to distinguish between demyelinating and axonal CMT^38^.

Another group of 52 molecularly unassigned patients was recruited from 138 index patients with a HNPP-like phenotype, after *PMP22* deletion was excluded by a RT-qPCR-based assay. The HNPP-like phenotype was defined by clinical manifestations compatible with the diagnostic guideline of HNPP regardless of genetic causes^39^. Sequencing analysis of the *PMP22* gene was applied to the 53 selected patients with demyelinating CMT and 52 cases with HNPP-like neuropathy. All the participants are of Han Chinese origin. Peripheral blood samples were collected after written informed consent was obtained from the participants or their parents on behalf of them for those younger than 18 years. This study conformed to the tenets of the Declaration of Helsinki, and all protocols of this study were approved by the Institutional Review Board of Taipei Veterans General Hospital. Written informed consent was obtained from all of the participants. Informed consent for publication of the images/video have been obtained from patient D.

### Mutation Analyses

Genomic DNA was extracted from peripheral blood cells using a standard protocol. The coding and flanking sequences of *PMP22* were amplified by PCR with intronic primers, and Sanger sequencing was then performed using the Big Dye 3.1 dideoxy terminator method (Applied Biosystems, Foster City, CA) on an ABI Prism 3700 Genetic Analyzer (Applied Biosystems). Amplicon sequences were compared with the reference *PMP22* coding genome (GRCh38, NM_000304.3). The pathogenicity of the identified variants was further ascertained by their absence in 500 neurologically healthy individuals of Han-Chinese origin recruited at our hospital and whole genome sequencing data of 997 Taiwanese controls available from Taiwan Biobank database (https://taiwanview.twbiobank.org.tw). The dbSNP databases (Build 149; https://www.ncbi.nlm.nih.gov/snp) and gnomAD (http://gnomad.broadinstitute.org) were also queried for the putative pathogenic variants^40^. Functional impacts of the *PMP22* variants were predicted *in silico* using PolyPhen-2 (http://genetics.bwh.harvard.edu)^22^, Mutation Taster (http://www.mutationtaster.org)^23^, and Combined Annotation Dependent Depletion (CADD) (http://cadd.gs.washington.edu)^24^. Evolutionary conservation of the mutation sites was analyzed by aligning amino-acid sequences of PMP22 orthologs from multiple species using the UniProt website (http://www.uniprot.org)^41^.

### *In vitro* analyses of PMP22 expression

#### Expression plasmids, cell culture and transfection

The WT cDNA clone of human *PMP22* (MGC 4588473) was purchased from Invitrogen (Carlsbad, CA). The full-length coding region of *PMP22* was cloned into pcDNA3.1/myc-His vector (Invitrogen) to generate the *PMP22* expression plasmid, which could produce a C-terminal myc-tagged WT PMP22 protein. To generate the mutant construct expressing myc-tagged Q86X PMP22 protein, the 5′-terminal 255 base pairs fragment of the WT *PMP22* coding sequence was subcloned in-frame into pcDNA3.1/myc-His vector. The mutations, c.124 T > C (p.C42R) and c.310delA (p.I104FfsX7), were separately introduced into the WT construct using QuikChange Site-Directed Mutagenesis kit (Stratagene, La Jolla, CA). Subsequently, the first 330 nucleotides of the c.310delA construct was subcloned in-frame into pcDNA3.1/myc-His vector to create the myc-tagged I104FfsX7 PMP22 expression plasmid.

Mouse neuroblastoma cell line Neuro-2a (ATCC® CCL-131^TM^) were maintained in Eagle’s Minimum Essential Medium (ATCC no.30-2003) containing 10% fetal bovine serum. Two kinds of rat neuronal Schwannoma cell lines, RSC96 (ATCC® CRL-2765^TM^) and RT4-D6P2T (ATCC® CRL-2768^TM^), were maintained in Dulbecco’s Modified Eagle’s Medium (ATCC no. 30-2002) containing 10% fetal bovine serum. All cells were cultured in a humidified 5% CO_2_ incubator at 37 °C. Transient transfections were performed using Lipofectamine 2000 (Invitrogen).

#### Western blot analyses and cycloheximide (CHX)-chase assays

Neuro-2a cells and RSC96 cells were transfected with WT *PMP22* or either one of the mutant *PMP22* expression plasmids (C42R, Q86X, or I104FfsX7). Forty-eight hours post-transfection, cells were lysed in radioimmunoprecipitation assay (RIPA) buffer supplemented with protease inhibitor cocktail (Merck Millipore, Darmstadt, Germany). The protein concentration was determined using a Bradford protein assay kit (Bio-Rad, Hercules, CA), and 50 μg of proteins from each lysate sample were used for Western blotting with c-Myc antibody (Santa Cruz Biotechnology, Dallas, TX). The protein bands were detected using a standard enhanced chemiluminescence method, and the densitometric analyses were performed using NIH ImageJ software.

To determine the stability of WT and mutant PMP22 proteins, CHX-chase assays were conducted with cells transfected with different *PMP22* constructs. Twenty-four hours after transfection, cells were trypsinized and re-seeded into 6-well culture plates. After additional 24 hours, CHX (Sigma-Aldrich, St. Louis, MO) was added to a final concentration of 0.1 mg/ml. Cell lysates were harvested at the indicated time points and subjected to Western blotting with the c-Myc antibody. Actin was used as a loading control. The ratios of PMP22 to actin were calculated densitometrically.

#### Immunofluorescence analyses

Forty-eight hours after transfection with the WT or mutant *PMP22* plasmids, Neuro-2a cells, RSC96 cells and RT4-D6P2T cells were fixed with 4% paraformaldehyde and then permeabilized in 0.2% tween-20. After blocking of non-specific binding with 5% bovine serum albumin, the cells were stained for PMP22 using the c-Myc antibody conjugated to Alexa Fluor 488 (Invitrogen) together with 4′,6-diamidino-2-phenylindole (DAPI) counter staining of cell nuclei. Immunofluorescent staining was examined under an Olympus FluoView FV10i confocal laser scanning fluorescence microscopy system with a 60X oil immersion objective (Olympus, Tokyo, Japan).

## Electronic supplementary material

Supplementary video

Supplementary material
