# Supplementary material for: Clinical and Molecular Characterization of PMP22 point mutations in Taiwanese patients with Inherited Neuropathy
Source: Sci Rep. 2017 Nov 10;7:15363. doi: 10.1038/s41598-017-14771-5 (PMC5681590; doi:10.1038/s41598-017-14771-5)

Clinical and Molecular Characterization of *PMP22* point mutations in Taiwanese patients with Inherited Neuropathy

Yi-Chu Liao, Pei-Chien Tsai, Thy-Sheng Lin, Cheng-Tsung Hsiao, Nai-Chen Chao, Kon-Ping Lin, Yi-Chung Lee

Supplementary Material

Supplementary Video Legend

The video of the patient D at age 26 years demonstrating pronounced atrophy of the leg muscles, bilateral foot drop and a steppage gait.

Supplementary Fig. S1. Atrophy of the intrinsic hand muscles as well as muscles in the feet and legs, and hammer toes in patient D harboring the *PMP22* p.I104FfsX7 mutation.

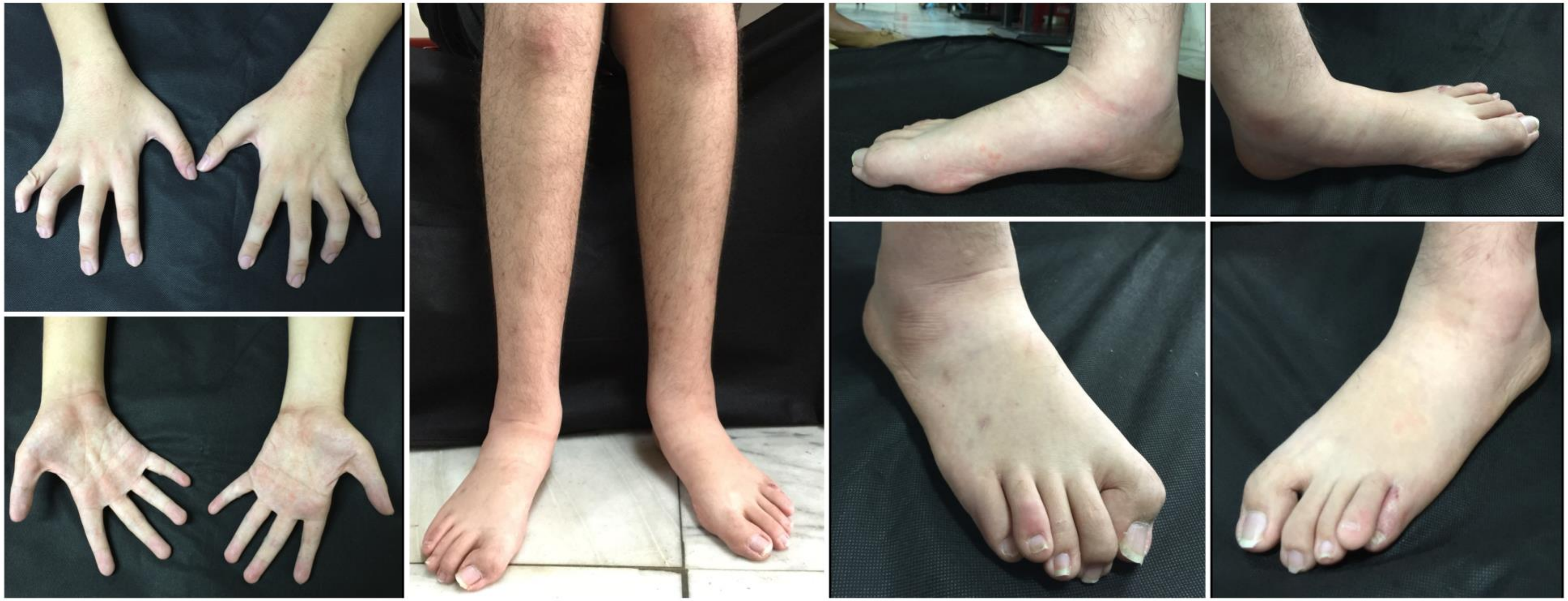

Supplementary Fig. S2. Western blot analysis of PMP22 protein expression in Neuro-2a cells showing I104FfsX7 mutant stuck at the interface between stacking gel and separating gel.

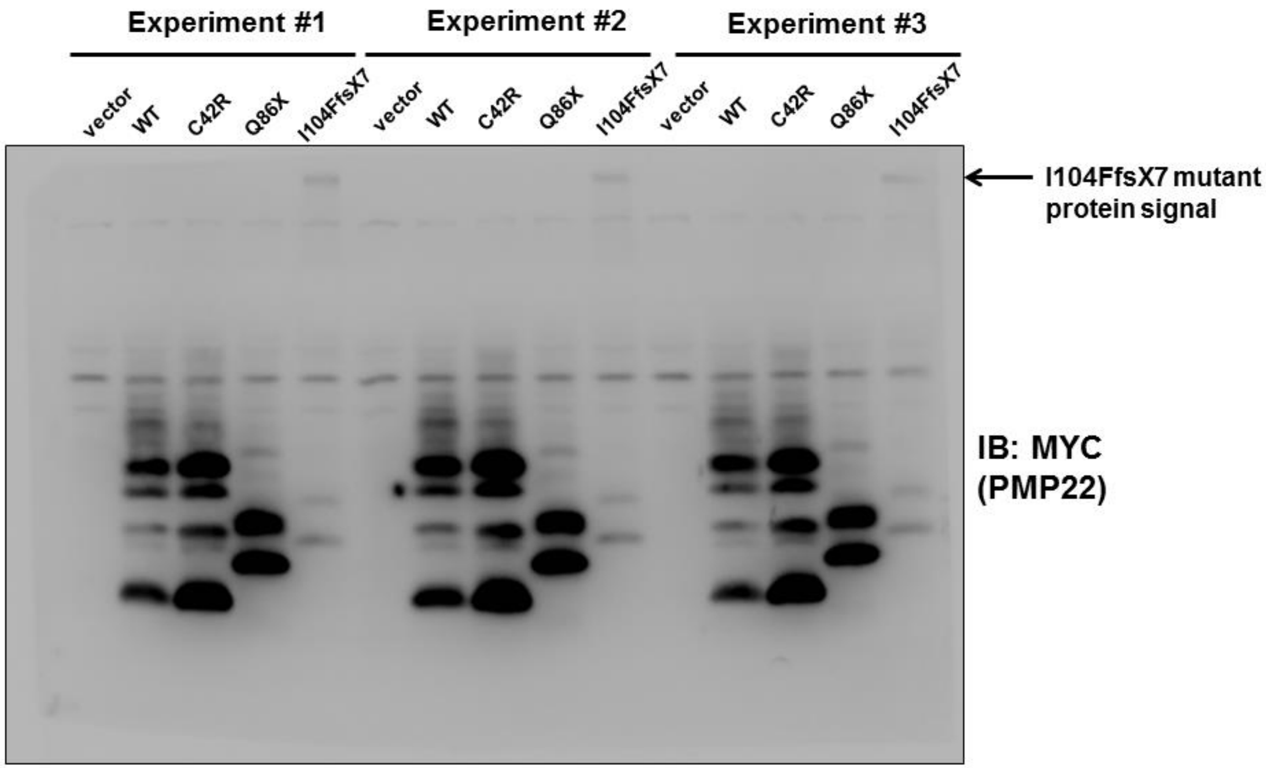

Supplementary Fig. S3. Cycloheximide (CHX)-chase assays showing different degradation rates of WT and mutant PMP22 proteins in RSC96 cells.

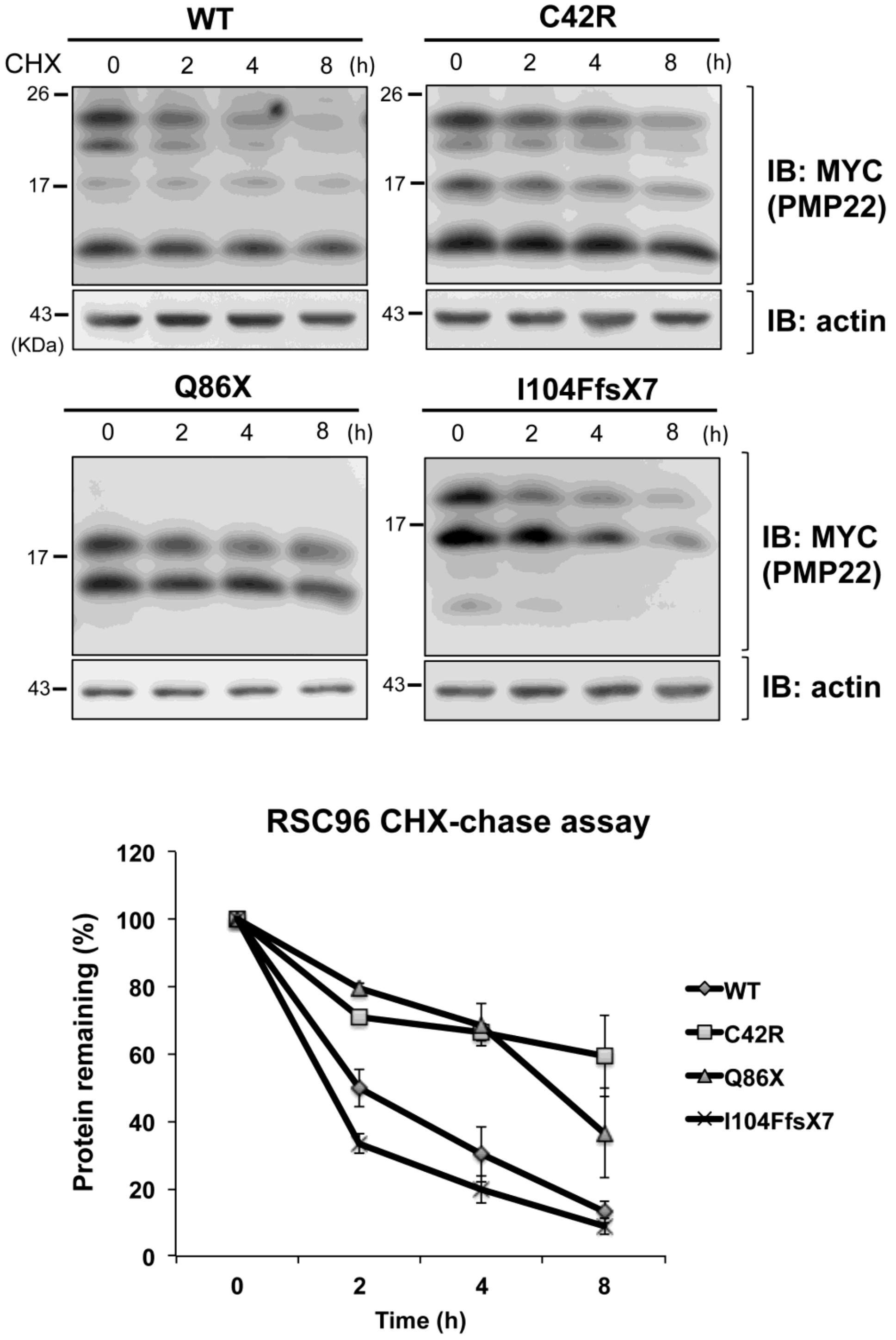

Supplement: Supplementary file 2 — Supplementary material [file 41598_2017_14771_MOESM2_ESM.pdf]
